# Ultra-Processed Food-Rich Dietary Proxies, Dietary-Pattern Context, and Cardiometabolic Outcomes in Spain: Weighted Evidence Consistent with Age Confounding and Possible Reverse-Causality Bias from the 2023 Spanish Health Survey

**DOI:** 10.3390/nu18162615

**Published:** 2026-08-10

**Authors:** Natividad Cuadrado-Corrales, José J. Zamorano-León, David Carabantes-Alarcón, Diana María Mérida, Andrés Bodas-Pinedo, Mario Chico-Rodríguez, Ana Lopez-de-Andres, Rodrigo Jiménez-Garcia, Enrique Redondo-González, Lucia Fuentes-Arroyo

**Affiliations:** 1 Department of Public Health and Maternal & Child Health, Faculty of Medicine, Universidad Complutense de Madrid, 28040 Madrid, Spain; mariancu@ucm.es (N.C.-C.); dcaraban@ucm.es (D.C.-A.); dmerida@ucm.es (D.M.M.); abodas@ucm.es (A.B.-P.); machic03@ucm.es (M.C.-R.); rodrijim@ucm.es (R.J.-G.); luciaifu@ucm.es (L.F.-A.); 2 Instituto de Investigación Sanitaria del Hospital Clínico San Carlos (IdISSC), 28040 Madrid, Spain; anailo04@ucm.es; 3 Department of Public Health and Maternal & Child Health, Faculty of Pharmacy, Universidad Complutense de Madrid, 28040 Madrid, Spain; 4 Department of Surgery, Faculty of Medicine, Universidad Complutense de Madrid, 28040 Madrid, Spain; eredon01@ucm.es

**Keywords:** ultra-processed food-rich proxies, fast food, processed meat, dietary pattern, obesity, hypertension, diabetes, cardiometabolic outcomes, reverse causality, ESdE 2023

## Abstract

Background/Objectives: Ultra-processed foods (UPFs) are linked to cardiometabolic disease, but cross-sectional surveys often underestimate or invert these associations due to age structure, diagnosis-related dietary changes, and reporting bias. This study explores the potential influence of these biases and examines cross-sectional associations between dietary proxies and prevalent cardiometabolic conditions using the most recent national data from Spain. Methods: We analyzed the 2023 Spanish Health Survey (n = 21,032). Body Mass Index (BMI) was derived from self-reported weight and height. Hypertension and diabetes were defined as self-reported medical diagnoses. Dietary items were mapped as UPF-rich proxies, processed-meat exposure, protective dietary markers, and beverage comparators. Weighted logistic models were adjusted for sociodemographic and lifestyle factors. Bias-probing analyses included stratification by self-rated health, exclusion of severe chronic comorbidities (n = 4107), and model-based beverage contrasts. Results: Prevalence reached 53.6% for excess weight, 23.9% for prevalent hypertension, and 7.4% for prevalent diabetes. Weekly fast-food consumption was consistently associated with excess weight (OR 1.20, 95% CI 1.07–1.35), obesity (OR 1.28, 95% CI 1.09–1.50), prevalent hypertension (OR 1.18, 95% CI 1.02–1.37), and prevalent diabetes (OR 1.33, 95% CI 1.10–1.61). Excluding participants with severe chronic comorbidities strengthened the fast-food association with prevalent diabetes (OR 1.46, 95% CI 1.17–1.81). Conclusions: Fast food emerged as the most consistent adverse dietary marker co-occurring across all prevalent outcomes, whereas processed meat was specifically associated with prevalent excess weight and obesity rather than prevalent cardiometabolic diagnoses. Stratified and sensitivity analyses provided empirical patterns consistent with age confounding and possible post-diagnosis dietary changes influencing cross-sectional associations, highlighting the need for cautious interpretation in public health surveillance.

## 1. Introduction

Cardiometabolic diseases are among the leading drivers of preventable morbidity and premature mortality worldwide. Excess adiposity, hypertension and type 2 diabetes frequently cluster within the same individuals and share behavioral, metabolic, inflammatory, and vascular pathways. Their coexistence is particularly relevant for public health because it amplifies vascular risk, encompassing both macrovascular complications, such as coronary heart disease, stroke, and peripheral artery disease, and microvascular damage affecting renal and retinal systems, which ultimately increases the demand for long-term clinical care [1]. In Spain, this burden represents a major public health concern; official data from the National Statistics Institute indicate that more than 55% of adults are living with excess weight (15.2% with obesity), while chronic conditions such as hypertension affect more than 20% of adults, showing a steady increase alongside type 2 diabetes over the last decade [2,3]. Crucially, historical trend analyses of the Spanish population since 1987 reveal that the prevalence of obesity has more than doubled, increasing from 7.3% to over 15.7% in recent years, with a persistent and alarming upward trajectory concentrated among youth and young adults aged 15 to 24 years [4].

Diet is a central modifiable determinant of cardiometabolic health. In Spain, the traditional Mediterranean dietary pattern has historically been characterized by high consumption of minimally processed plant foods, legumes, fish, and olive oil. However, population dietary behaviors have progressively shifted towards greater availability and intake of industrially formulated, energy-dense, and nutrient-poor products [5]. Recent evidence suggests that the dietary share of ultra-processed foods (UPFs) in Spain has increased substantially over recent decades, with their contribution rising from 11% to approximately 32% of total daily energy intake [6]. UPFs, as defined by the NOVA classification, are industrial formulations typically characterized by high palatability, long shelf life, and frequent addition of refined starches, sugars, fats, salt, flavor enhancers, emulsifiers, and other additives [7]. Higher UPF intake has been associated with adiposity, diabetes, hypertension, cardiovascular risk, and mortality in prospective and meta-analytic evidence [8,9,10,11].

Nevertheless, the measurement and interpretation of UPF exposure in national health surveys remain challenging. Many population surveys do not contain sufficient information to apply a complete NOVA classification. Instead, they include selected food-frequency items such as sugar-sweetened beverages (SSB), sweets/bakery products, fast food, and salty snacks. These items can be interpreted as food-frequency proxies, but not as a complete quantitative estimate of total NOVA-defined ultra-processed food intake or daily dietary energy share. Instead, they function as discrete UPF-proxy indicators. A transparent distinction between total UPF exposure and UPF-proxy indicators is therefore essential to avoid exposure misclassification and overinterpretation [12,13].

A second challenge is temporal interpretation. In cross-sectional surveys, current dietary intake and prevalent disease are measured at the same time. Participants diagnosed with obesity, hypertension, or diabetes may reduce SSB, fast food, salty snacks, or other foods perceived as unhealthy after receiving medical advice. In addition, individuals with chronic disease or excess weight may differentially underreport socially undesirable foods. These mechanisms can produce inverse crude associations between unhealthy foods and cardiometabolic diagnoses, even when the long-term etiological association is adverse [14,15].

The 2023 Spanish Health Survey provides a nationally representative framework to examine this methodological problem in a contemporary public health context [2]. While the UPF construct encompasses a wide range of industrial formulations, national health surveys such as the ESdE 2023 [2] often lack the granularity required for a full NOVA-based categorization. In such contexts, identifying specific indicators such as fast-food item responses becomes essential, as it serves as a concentrated and interpretable UPF-rich proxy. This approach allows researchers to capture high industrial exposure and dietary risk in samples where total UPF energy contribution cannot be directly calculated.

Accordingly, this study aimed to: (1) describe the weighted cardiometabolic and dietary profile of the Spanish population aged 15 years and older; (2) map ESdE food-frequency items according to their interpretability as UPF-rich proxies, processed-meat proxies, and protective dietary markers; (3) evaluate associations between UPF-proxy burden, processed meat, fast food, and cardiometabolic outcomes; and (4) assess evidence consistent with hypothesized bias mechanisms through self-rated health stratification, exclusion of severe chronic comorbidity, an age-scope sensitivity analysis excluding participants aged 15–17 years, and a model-based, frequency-equivalent beverage contrast [16].

## 2. Materials and Methods

### 2.1. Study Design and Data Source

This was a weighted cross-sectional analysis of the adult file of the 2023 Spanish Health Survey (Encuesta de Salud de España, ESdE 2023). The ESdE is a national health survey jointly developed by the Spanish Ministry of Health and the National Statistics Institute to provide harmonized information on health status, healthcare use, health determinants, and sociodemographic characteristics [2]. The adult questionnaire targets individuals aged 15 years and older residing in main family dwellings.

### 2.2. Analytical Sample

The adult microdata file included 21,032 respondents. Analyses used all participants with valid information for each outcome, exposure, and model-specific covariate set. The complete-case flow and the number of participants retained after applying outcome-, exposure-, and covariate-specific validity criteria are reported in Table S1. The adult sampling factor was used for weighted descriptive estimates and weighted regression models. Because public variables for primary sampling units and strata were not available in the working file, confidence intervals were estimated using normalized sampling weights and robust sandwich covariance; these should be interpreted as approximate rather than fully design-based intervals [17].

Missing data were handled using a concurrent complete-case approach for the multivariable analyses. To ensure strict comparability between the primary multivariable models and the exploratory beverage-contrast sensitivity analyses, a consistent outcome-specific complete-case definition was applied. Consequently, the analytical sample was restricted to participants with non-missing data for the relevant outcome, all primary dietary predictors, adjustment covariates, and the natural fruit/vegetable juice comparator used in the secondary beverage-contrast analyses. Natural fruit/vegetable juice was not entered as a predictor in the primary multivariable models. However, missingness in this variable was included in the complete-case filtering step to ensure that the primary analyses and the beverage-contrast analyses were estimated using identical outcome-specific analytical samples. Because data availability differed across outcomes, analytical samples ranged from 17,122 to 17,952 participants. Table S1 reports variable-specific missingness and the complete-case flow for each model. Table S2 compares selected sociodemographic, lifestyle, and health characteristics between included participants and those excluded because of missing data.

### 2.3. Cardiometabolic Outcomes

All outcomes were evaluated as prevalent conditions at the time of the survey. Specifically, body mass index (BMI) was based on the official ESdE BMI category variable, where prevalent excess weight was defined as being overweight or obese (BMI ≥ 25 kg/m^2^). Obesity was analyzed as a secondary adiposity outcome (BMI ≥ 30 kg/m^2^).

Regarding non-adiposity outcomes, prevalent hypertension was defined as a self-reported medical diagnosis of arterial hypertension, and prevalent diabetes as a self-reported medical diagnosis of diabetes mellitus.

In addition to individual outcomes, a dual cardiometabolic phenotype was specified as the simultaneous presence of excess weight (BMI ≥ 25 kg/m^2^) and self-reported prevalent hypertension.

From a clinical perspective, this composite endpoint represents a pragmatic proxy for adiposity-driven vascular resistance and early cardiometabolic strain. Because overweight/obesity and elevated blood pressure frequently cluster prior to formal clinical progression toward complex multimorbidity, monitoring this dual profile provides high surveillance relevance for primary prevention in adult populations [1].

### 2.4. Dietary Variables and NOVA Compatibility

Because the survey was not designed as an energy-calibrated dietary recall, available dietary variables capture consumption frequency rather than absolute grams, portion sizes, or total energy contribution from ultra-processed foods. The dietary framework separated UPF-rich proxies, processed-meat exposure, protective dietary markers, and ambiguous beverage comparator. Four variables were retained as primary UPF-rich proxies: sweets/bakery products, SSB, fast food, and salty snacks/fried potatoes. Weekly or more frequent intake was defined as any response category corresponding to ≥1 time/week, including 1–2 times/week, 3–6 times/week, and daily consumption. These four indicators were summed to create a UPF-proxy burden score ranging from 0 to 4 and categorized as 0–1, 2, and 3–4 proxies. This score was used to capture cumulative exposure to UPF-rich food-frequency groups, not total NOVA-defined ultra-processed food intake. The detailed analytical mapping of all selected ESdE 2023 dietary items, along with their specific variables, classifications, and corresponding NOVA compatibility, is fully detailed in Table 1.

To contextualize diet quality, we derived a protective dietary score based on four markers: daily fruit intake, daily vegetable intake, weekly or more frequent legume intake, and weekly or more frequent fish intake. The score ranged from 0 to 4 and was categorized as 0–1, 2, and 3–4 markers. The protective dietary score combines daily markers (fruit, vegetables) and weekly markers (legumes, fish) into a pragmatic additive score. A limitation of this operationalization is that item intake frequencies differ across components, and the fish item does not differentiate between fresh fish (NOVA 1) and processed/canned fish (NOVA 3/4), which may introduce exposure misclassification.

Processed meat was analyzed as a secondary processed-meat proxy because the ESdE item does not distinguish minimally processed, processed, and ultra-processed formulations. Because the ESdE survey item aggregates both traditionally cured meats (NOVA 3) and industrially formulated ultra-processed meat products containing cosmetic emulsifiers and preservatives (NOVA 4), exposure misclassification is possible, which may influence the magnitude of observed associations with adiposity outcomes. Natural fruit/vegetable juice was retained as an ambiguous descriptive beverage comparator. It was used for population characterization and the prespecified beverage-contrast sensitivity analysis but was not entered into the primary multivariable models to preserve model parsimony and avoid overadjustment by a heterogeneous beverage item. However, it is important to note that commercial packaged juices frequently undergo industrial reconstitution, sugar addition, or ultra-processing (NOVA 4), whereas freshly squeezed juices represent minimally processed items (NOVA 1). Because the ESdE item aggregates both types without distinction, its NOVA status is inherently mixed and ambiguous.

In the absence of quantitative portion-size data in national health surveillance microdata, dietary proxies were operationalized into pragmatic additive scores. Equal weighting (1 point per component indicator) was applied to construct both the UPF-proxy burden score (0–4) and the protective dietary score (0–4). This unweighted additive approach was selected a priori to prevent post-hoc, data-driven optimization and sample-specific overfitting. Cutoff thresholds for individual food components were established based on epidemiological intake distributions in the survey and alignment with prior surveillance studies.

This mapping supports transparent interpretation of food-frequency items, clarifies the analytical role of each dietary variable, and prevents overinterpretation of survey items as a strict NOVA classification. Natural fruit/vegetable juice was retained as a descriptive comparator and used only in the beverage-contrast sensitivity analysis; it was not included in the primary regression tables.

Among the dietary items analyzed, fast-food item response was prioritized as the most interpretable UPF-rich meal-level proxy, while acknowledging that the item is composite and cannot be equated with total NOVA-defined UPF intake. Compared with other food-frequency items, fast food is likely to capture a higher-exposure industrial dietary pattern, but the ESdE variable does not provide information on ingredients, preparation methods, portion size, or total energy contribution. Therefore, findings for fast-food item response should be interpreted as associations with a UPF-rich dietary proxy rather than as estimates of total ultra-processed food intake.

### 2.5. Covariates

The fully adjusted models included age, age squared, sex, educational level, occupational social class, leisure-time physical activity, smoking status, and alcohol intake. Educational level was grouped as low, medium, and high. Occupational social class was grouped as I-II, III–IV, and V–VI. Leisure-time physical activity was categorized as sedentary, occasional, or active; smoking as never, former, or current; and alcohol intake as none/former, occasional, or regular. Dietary-pattern context was incorporated through the protective dietary score and, where relevant, processed-meat exposure.

### 2.6. Statistical Analysis

Categorical variables were described as unweighted counts and weighted percentages. Weighted 95% confidence intervals for prevalence estimates were calculated using a Kish effective-sample-size approximation [18]. To ensure proper weighting, logistic regression models were estimated with normalized adult sampling weights (rescaled so that the sum of weights equaled the complete-case analytical sample size). This ensured that point estimates accurately reflected population proportions while preventing artificially inflated statistical precision. Furthermore, sandwich standard error estimators (Huber–White) were applied to obtain heteroskedasticity-consistent regression standard errors [17]. Regarding survey design constraints, because primary sampling unit (PSU) and stratum variables are omitted from the ESdE public-use microdata for confidentiality reasons, variance estimation could not incorporate complex design features such as spatial clustering. Therefore, reported confidence intervals represent weighted, model-based consistent estimates rather than fully complex design-based survey estimates.

For the primary multivariable analysis, weighted multivariable logistic regression models were estimated to calculate ORs for the prevalence of each cardiometabolic outcome (prevalent excess weight, prevalent obesity, prevalent hypertension, prevalent diabetes, and prevalent dual profile). For each outcome, crude weighted odds ratios were first calculated for each food proxy. To illustrate how covariate adjustment changed selected diet–cardiometabolic associations, we fitted sequential weighted logistic regression models for a set of bias-sensitive exposure–outcome pairs. Model 0 included only dietary exposure. Model 1 additionally adjusted for age, age squared, and sex. Model 2 further adjusted for socioeconomic position, including educational level and occupational social class. Model 3 additionally adjusted for lifestyle factors, including leisure-time physical activity, smoking status, and alcohol intake. Model 4 corresponded to the fully adjusted specification used in the main analyses, additionally incorporating broader dietary-pattern context through mutual adjustment for the relevant dietary proxies and the protective dietary score. For each exposure–outcome pair, all sequential models were estimated within the same complete-case sample used for the fully adjusted model, ensuring that changes in odds ratios reflected covariate adjustment rather than differences in analytical sample size (Table S3). The final model specifications used for Tables 4 and 5 were defined as follows. Table 4 used a construct-level dietary model including the UPF-proxy burden score, processed meat, protective dietary score, age, age squared, sex, educational level, occupational social class, leisure-time physical activity, smoking status, and alcohol intake. Table 5 used an individual-proxy dietary model including sugar-sweetened beverages, sweets/bakery products, fast food, salty snacks/fried potatoes, processed meat, protective dietary score, age, age squared, sex, educational level, occupational social class, leisure-time physical activity, smoking status, and alcohol intake. Natural fruit/vegetable juice was not included as an exposure or covariate in either Table 4 or Table 5; it was used only in the descriptive dietary profile and in the exploratory model-based beverage-contrast sensitivity analysis. However, missingness in the natural-juice variable was included in the concurrent complete-case filtering step to preserve identical outcome-specific analytical samples across the primary and beverage-contrast analyses.

Finally, to assess the sensitivity of the age specification, sensitivity specifications compared continuous quadratic age modeling against 10-year categorical brackets and restricted cubic splines with 4 knots to ensure adequate control of age structure (Table S4). Additionally, sequential adjustment models were fitted to track covariate displacement and evaluate potential negative confounding across adjustment steps (Table S3).

Multivariable models adjusted for age, age squared, sex, education, occupational social class, leisure-time physical activity, smoking, and alcohol intake. Sandwich standard error estimators were used for regression-based confidence intervals [17]. Pairwise, weighted correlations among dietary indicators and variance-inflation factors for dietary predictors were calculated as collinearity diagnostics and are reported in Tables S5 and S6. Statistical interpretation prioritized magnitude, direction, and epidemiological plausibility rather than *p*-values alone.

The primary models estimated associations between the UPF-proxy burden score and cardiometabolic outcomes, adjusting for processed meat, protective dietary score and sociodemographic/lifestyle covariates.

Secondary models estimated associations for individual food proxies simultaneously. Consequently, models including multiple dietary indicators should therefore be interpreted as conditional associations within a broader dietary-pattern context, not as total effects of each food group.

Regarding statistical variance, because public-use ESdE microdata omit PSU and stratum identifiers to protect confidentiality, complex survey variance estimation relied on normalized sampling weights and robust sandwich covariance estimators (Huber–White), producing weighted model-based consistent estimates.

In addition, a frequency-category analysis for fast food evaluated four intake levels (less than weekly/never, 1–2 times/week, 3–6 times/week, and daily) to examine risk patterns across consumption frequencies. Because multiple exposure–outcome pairs were evaluated, *p*-values were interpreted descriptively alongside parameter precision (95% CIs), effect magnitude, biological plausibility, and consistency across models rather than as independent confirmatory tests.

To probe for potential bias arising from post-diagnosis dietary modification or disease-related reverse causality, sensitivity analyses were conducted by excluding participants with severe chronic comorbidities. This restriction included major clinically diagnosed conditions: myocardial infarction, angina or coronary heart disease, stroke, malignant tumors, stomach or duodenal ulcers, cirrhosis or severe liver dysfunction, and chronic kidney failure. These specific conditions were selected because they represent major organic pathologies that trigger formal clinical dietary interventions, strict medical restrictions, or substantial systemic illness, which are highly prone to altering routine food choices and masking primary population-level exposure gradients in cross-sectional settings.

Fully adjusted multivariable models served as primary analyses, whereas stratified and sensitivity specifications represented secondary bias-probing evaluations. Formal multiplicity corrections were not applied to prevent type II errors; instead, interpretation prioritized effect magnitude, 95% confidence intervals, and overall pattern consistency over isolated *p*-values.

### 2.7. Assessment of Collinearity Among Dietary Predictors

Because several dietary variables were conceptually related, we assessed the degree of overlap between dietary predictors before interpreting the multivariable models. We calculated a weighted correlation matrix including the UPF-proxy burden score, individual UPF-rich indicators, processed meat, and the protective dietary score and its components (Table S5). We also estimated variance-inflation factors for the dietary predictors entered in the fully adjusted models. Correlation coefficients were used to describe construct overlaps, whereas VIF values were used to assess potential multicollinearity (Table S6). VIF values below 2 were interpreted as evidence against problematic collinearity among dietary predictors.

### 2.8. Bias-Probing Analyses

To examine whether inverse associations were compatible with possible post-diagnosis behavioral change or reporting bias, we conducted several additional bias-probing and sensitivity analyses (Table S7). First, we stratified the fully adjusted models by self-rated health, contrasting respondents reporting good or very good health with those reporting fair, poor, or very poor health. This stratification was intended as an indirect bias-probing analysis, not as a definitive separation of participants with and without post-diagnosis dietary change. Self-rated health was used as a proxy for overall perceived health status and potential health-related behavioral adaptation, recognizing that participants reporting good or very good health may still have chronic diagnoses, treatment-related dietary advice, or recent dietary changes.

Second, we repeated the fully adjusted models after excluding respondents with severe chronic comorbidity. Severe comorbidity was defined as a self-reported medical diagnosis of myocardial infarction, angina/coronary disease, other heart disease, stroke, malignant tumors, stomach/duodenal ulcer, cirrhosis/liver dysfunction, or kidney problems, based on the ESdE chronic-disease module.

Third, because the ESdE public-use file lacks a water-consumption frequency variable, we evaluated an exploratory, non-isocaloric, frequency-equivalent beverage contrast comparing natural juice with SSB intake. In multivariable models incorporating SSB and natural juice frequency categories simultaneously, this statistical contrast was calculated as exp(β_natural juice_-β_SSB_). We explicitly clarify that this parameter represents a frequency-equivalent model contrast derived from simultaneous regression coefficients, not an observed dietary replacement, an isocaloric substitution, or a causal beverage exchange.

Fourth, to confirm that standard adult BMI cut-offs did not introduce misclassification bias, a sensitivity analysis excluded respondents aged 15–17 years (n = 393) (Table S7).

Fifth, to verify that non-linear age structure was adequately controlled and less vulnerable to residual confounding by age structure, we re-estimated all primary multivariable models replacing the continuous quadratic age term (age + age^2^) with alternative age parameterizations: 10-year categorical age groups and restricted cubic splines with 4 knots placed at the 5th, 35th, 65th, and 95th percentiles (Table S4).

## 3. Results

### 3.1. Weighted Population: Cardiometabolic and Dietary Profile

The analytical sample included 21,032 individuals aged 15 years or older. After accounting for complete cases across outcome-specific covariate matrices, the final analytic sample sizes ranged from n = 17,122 (for the dual phenotype) to n = 17,952 (for diabetes) (see Table S1 for complete item-specific missingness details). Overall, the weight prevalence was 53.6% for excess weight, 14.7% for obesity, 23.9% for prevalent hypertension, 7.4% for prevalent diabetes, and 17.1% for the combined hypertension-plus-excess-weight phenotype.

Regarding the dietary behaviors operationally defined in Section 2.4, protective markers were common but uneven: 53.0% reported daily fruit intake, 32.6% reported daily vegetable intake, and only 25.5% reported both fruit and vegetables daily. Weekly or more frequent intake was 88.5% for legumes and 82.2% for fish; 52.0% had a high protective dietary score. UPF-rich proxies were also frequent: 71.1% consumed sweets/bakery products weekly or more, 34.8% SSB, 38.0% fast food, and 43.7% salty snacks. A high UPF-proxy burden score was present in 34.1% of the weighted population. Processed meat showed very high weekly prevalence (76.5%), while natural juice was reported by 39.1% (Table 2).

### 3.2. Age-Patterned Dietary Exposure and Cardiometabolic Burden

Cardiometabolic outcomes and dietary exposures showed strongly opposing age gradients (Table 3). Prevalent hypertension increased from 0.4% at ages 15–17 to 60.4% at ages 75 years and older, and prevalent diabetes increased from 0.6% to 23.8%. By contrast, a high UPF-proxy burden score decreased from 62.1% to 8.4%, and fast-food item response decreased from 65.8% to 9.6%. Processed-meat intake also declined with age, although less steeply, whereas a high protective dietary score increased from 41.4% in the youngest group to 67.0% in those aged 75 years and older. This reversal provides empirical support for age confounding in crude diet–disease associations.

Sequential models comparing crude, age-adjusted, socioeconomic-adjusted, lifestyle-adjusted, and fully adjusted estimates for selected exposure–outcome pairs are shown in Table S3 Tracking this association across sequential adjustment models revealed a clear negative confounding (suppression) pattern: the fast-food estimate for prevalent diabetes shifted systematically from crude OR 0.58 (95% CI 0.48–0.70) to age-adjusted OR 0.86 (95% CI 0.71–1.04), SES-adjusted OR 1.09 (95% CI 0.89–1.33), lifestyle-adjusted OR 1.25 (95% CI 1.02–1.52), and fully adjusted OR 1.33 (95% CI 1.10–1.61) (Table S3).

### 3.3. UPF-Proxy Burden, Processed Meat, and Protective Dietary Score

In fully adjusted models, individuals with a high UPF-proxy burden score (3–4 proxies vs. 0–1) exhibited higher odds of excess weight (OR 1.16, 95% CI 1.05–1.28) (Table 4). Regarding specific components, weekly processed meat intake was positively associated with excess weight (OR 1.18, 95% CI 1.06–1.31) and obesity (OR 1.25, 95% CI 1.09–1.43), while displaying no consistent cross-sectional associations with prevalent hypertension or diabetes. The protective dietary score was inversely associated with excess weight (OR 0.93 per category, 95% CI 0.89–0.97), but not consistently with diagnosed cardiometabolic outcomes. The inverse associations between UPF-proxy burden and prevalent diabetes are more plausibly compatible with diagnosis-related restriction, differential reporting, or residual reverse causality than with inverse association (Table 4).

### 3.4. Individual Dietary Proxies in Fully Adjusted Models

When individual dietary proxies were included simultaneously, fast food remained the most consistent adverse marker (Table 5).

Because these models are mutually adjusted for several dietary indicators, the estimates should be interpreted as conditional associations rather than total dietary effects. Weekly or more frequent fast-food item response was associated with excess weight (OR 1.20, 95% CI 1.07–1.35), obesity (OR 1.28, 95% CI 1.09–1.50), prevalent hypertension (OR 1.18, 95% CI 1.02–1.37), prevalent diabetes (OR 1.33, 95% CI 1.10–1.61), and the dual profile (OR 1.22, 95% CI 1.04–1.43) in fully adjusted models (Table 5). SSB were positively associated with excess weight and obesity but remained inversely associated with prevalent diabetes, consistent with diagnosis-related avoidance. Processed meat was positively associated with excess weight and obesity, whereas sweets/bakery products remained inversely associated with several outcomes, likely reflecting residual dietary adaptation after clinical diagnosis, reporting bias, or category heterogeneity.

### 3.5. Fast-Food Frequency-Category Analysis

Evaluating fast-food item-response frequency categories showed positive associations with several prevalent cardiometabolic outcomes, particularly for the 3–6 times/week category (Table 6).

Compared with less than weekly or no intake, consumption 1–2 times per week was associated with higher odds of prevalent hypertension, prevalent diabetes, and dual profile, while consumption 3–6 times per week showed stronger associations across all outcomes. The daily category showed wider confidence intervals and non-monotonic estimates for some outcomes, consistent with lower precision in this smaller exposure group.

For daily fast-food intake, estimates showed wider confidence intervals and non-monotonic patterns across outcomes (e.g., hypertension: OR 0.90, 95% CI 0.49–1.64), with strongest associations for prevalent obesity and diabetes. These results support evaluating fast food across distinct intake-frequency categories rather than assuming a continuous or strictly monotonic dose-response relationship, given the non-monotonic estimates and wider confidence intervals observed in the daily category.

### 3.6. Dietary-Predictor Correlations and VIF Results

Weighted dietary correlations and VIF diagnostics did not indicate problematic multicollinearity among dietary predictors. Correlations were strongest between the UPF-proxy burden score and its individual components, as expected, whereas correlations between UPF-rich indicators and the protective dietary score were weak (Table S5). In multivariable models, variance-inflation factors for all primary dietary predictors ranged between 1.04 (protective dietary score) and 1.52 (fast food frequency), supporting the absence of problematic multicollinearity (all VIFs < 2.0; Table S6).

### 3.7. Empirical Bias-Probing Analyses

Exclusion of participants aged 15–17 years produced comparable adjusted estimates for weekly fast-food item frequency across prevalent cardiometabolic outcomes, indicating that the inclusion of this small age group did not materially influence the main findings (Table S7).

In addition, the supplementary analyses provided empirical support for the hypothesized bias structure (Table 7). Specifically, stratification by self-rated health status revealed significant effect-measure modification for unhealthy dietary proxies. Among respondents reporting good or very good health, estimates for SSBs were close to the null for prevalent hypertension (OR 0.99, 95% CI 0.87–1.13) and the dual profile (OR 1.01, 95% CI 0.87–1.18).

Conversely, among respondents reporting fair, poor, or very poor health, inverse associations were significantly more pronounced (e.g., SSBs and prevalent hypertension: OR 0.76, 95% CI 0.63–0.91; *p*-interaction = 0.012). These findings are consistent with possible post-diagnosis dietary change or differential reporting among individuals with worse self-rated health.

However, for prevalent diabetes, the inverse SSB association persisted even in the good/very good health stratum (OR 0.49, 95% CI 0.38–0.65; *p*-interaction = 0.124), consistent with diabetes-specific dietary adaptation or reporting patterns that may occur independently of overall health perception. Sweets/bakery products remained inversely associated with several outcomes across both strata, consistent with residual reverse causality, selective restriction, or differential reporting.

Exclusion of participants aged 15–17 years (n = 393) produced virtually identical odds ratios across all cardiometabolic outcomes compared to the primary sample (e.g., weekly fast food and excess weight: OR 1.20, 95% CI 1.07–1.35; weekly fast food and prevalent diabetes: OR 1.34, 95% CI 1.11–1.61) (Table S7).

Sensitivity analyses evaluating alternative age specifications (10-year categorical age groups and restricted cubic splines with four knots) yielded virtually identical odds ratios and 95% confidence intervals across all primary dietary exposures and cardiometabolic outcomes compared to the quadratic continuous age specification (e.g., weekly fast food and prevalent diabetes: quadratic age OR 1.33, 95% CI 1.10–1.61; categorical age OR 1.32, 95% CI 1.09–1.60; restricted cubic splines OR 1.33, 95% CI 1.10–1.61) (Table S4), supporting the adequacy of the quadratic age specification for the observed age structure.

Furthermore, sensitivity analyses evaluating alternative score formulations supported the interpretation of the primary analyses, particularly for adiposity-related outcomes (Table S8). Modeling the UPF-proxy burden score as a continuous variable (0–4 scale) showed positive per-unit associations with excess weight (OR 1.07 per unit increase, 95% CI 1.03–1.11), obesity (OR 1.09 per unit increase, 95% CI 1.04–1.15), and the dual profile (OR 1.06 per unit increase, 95% CI 1.01–1.12). Similarly, evaluating component intake frequencies as ordinal variables (never/rarely to daily) yielded patterns consistent with the primary binary models (Table S8).

The persistence of an inverse association between SSB and prevalent diabetes (OR 0.49, 95% CI 0.38–0.65) even among respondents with good or very good self-rated health is consistent with residual post-diagnosis dietary change, post-diagnosis dietary restriction, or differential reporting, rather than with a biological inverse association. Furthermore, excluding participants with severe chronic conditions unmasked a stronger positive association for diabetes (OR 1.46 vs. OR 1.33 in the primary model), a pattern compatible with the possibility that post-diagnosis dietary restriction and related reporting mechanisms may attenuate cross-sectional estimates. However, because these analyses remain cross-sectional and proxy-based, they should be interpreted as bias-probing evidence rather than as causal confirmation.

Regarding beverage comparisons, the exploratory, non-isocaloric, frequency-equivalent beverage contrast comparing weekly natural juice with weekly SSB intake yielded lower odds of excess weight (OR 0.89, 95% CI 0.80–0.99) and obesity (OR 0.81, 95% CI 0.70–0.95), but a counter-intuitive positive association for prevalent diabetes (OR 1.54, 95% CI 1.22–1.95). This paradoxical diabetes estimate underscores the limitations of modeling cross-sectional beverage contrasts, reinforcing that these statistical comparisons should not be interpreted as causal dietary substitutions.

Additionally, the restricted analysis excluding 4107 participants with severe chronic comorbidities (e.g., heart disease, stroke, cancer) yielded patterns consistent with the primary findings. In this healthier subgroup, fast-food intake remained positively associated with all outcomes, and the association with prevalent diabetes strengthened from OR 1.33 in the primary model to OR 1.46 (95% CI 1.17–1.81). Similarly, associations for processed meat with prevalent excess weight (OR 1.15) and obesity (OR 1.31) remained significant and consistent with the main analyses (Supplementary Table S7).

## 4. Discussion

### 4.1. Principal Findings

This nationally representative cross-sectional analysis of the ESdE 2023 provides two complementary contributions to nutritional epidemiology and public health surveillance in Spain by evaluating the co-occurrence of dietary proxies and prevalent cardiometabolic conditions. First, it confirms a substantial cardiometabolic burden in the population aged 15 years and older: 53.6% had excess weight, 14.7% had obesity, 23.9% reported hypertension, 7.4% reported prevalent diabetes, and 17.1% had the combined hypertension-plus-excess-weight phenotype. While these estimates are coherent with national Spanish surveillance, their interpretation must account for the self-reported nature of the ESdE outcomes.

In comparison with clinically measured studies, the obesity estimate is close to the self-reported national trend described by Feijoo et al., who documented an increase in adult obesity in Spain from 7.3% in 1987 to approximately 15.7% in 2020, while measured anthropometric studies such as ENRICA have generally produced higher absolute estimates because weight and height were directly assessed [4,19]. Similarly, the 7.4% self-reported prevalent diabetes prevalence observed here should be interpreted as diagnosed prevalent diabetes rather than total prevalent diabetes burden; the Di@bet.es study, which included biochemical assessment, estimated total prevalent diabetes prevalence at 13.8%, including both known and previously undiagnosed prevalent diabetes [3,20]. Likewise, the hypertension estimate of 23.9% is also lower than the 42.6% prevalence reported in Di@bet.es using measured blood pressure and treatment information, again reflecting the difference between self-reported diagnosis and clinically ascertained hypertension [21]. Therefore, the ESdE estimates are externally plausible for a self-reported national health survey, but they should not be equated with biomarker- or examination-based prevalence estimates.

Among the evaluated ultra-processed food proxies, weekly fast-food consumption emerged as the most consistent observed proxy signal across cardiometabolic outcomes in fully adjusted models, showing its strongest links to obesity and prevalent diabetes alongside a more modest association with hypertension. This pattern is more consistent with stronger cross-sectional associations for adiposity and diabetes-related outcomes than for hypertension, although mechanisms cannot be inferred from these data. However, given the cross-sectional nature of the data and potential residual confounding, this finding should be interpreted as a prominent population-level association rather than an unconfounded protective or causal effect. In contrast, weekly processed meat demonstrated an outcome-specific relationship: it was positively associated with adiposity markers (excess weight and obesity) but showed no consistent cross-sectional association with prevalent hypertension or diabetes. This outcome specificity indicates that processed meat intake in this survey context tracks primarily with adiposity indicators rather than distinct cardiometabolic clinical diagnoses.

Furthermore, the composite dual profile yielded odds ratios that were generally intermediate between those for isolated excess weight and isolated hypertension, reflecting the combined metabolic and vascular burden of this high-risk phenotype without introducing significant statistical divergence. Likewise, the UPF-proxy burden score was mainly associated with excess weight, while the protective dietary score showed an inverse association with excess weight. These findings suggest that specific high-risk dietary markers may be more informative than treating all food-frequency items as equivalent UPF indicators.

Likewise, the model-based beverage contrast yielded an inverse association with excess weight (OR 0.89, 95% CI 0.80–0.99) but a paradoxical positive association with prevalent diabetes (OR 1.54, 95% CI 1.22–1.95). This paradoxical diabetes estimate is compatible with possible post-diagnosis dietary restriction that affects sugar-dense beverages generally: individuals diagnosed with diabetes receive clinical guidance to restrict both SSBs and fruit juices, leading to concurrent reductions in both items and rendering cross-sectional statistical contrasts uninterpretable as causal substitutions.

### 4.2. Why Crude Inverse Associations Matter

A central message of this study is that inverse crude associations between unhealthy foods and chronic diagnoses should not be interpreted as protective effects. Such an interpretation would be biologically implausible and inconsistent with the broader literature linking UPF-rich dietary patterns, processed meat, and SSBs with adverse cardiometabolic health [8,22,23,24]. Instead, these inverse estimates should be understood as an epidemiological warning.

Three mutually reinforcing mechanisms probably explain this inverse or attenuated cross-sectional estimates. First, age is a strong structural confounder in the ESdE data: younger respondents reported higher consumption of fast food and SSBs, whereas hypertension, diabetes, and the dual cardiometabolic phenotype increased markedly with age. Furthermore, socioeconomic position (SEP) exerts a complex, dual influence on national health surveillance. SEP influences not only dietary access and intake patterns but also healthcare utilization, frequency of clinical screening, and the probability of receiving a formal medical diagnosis. Simultaneously, higher SEP is associated with greater health literacy and social desirability bias in dietary reporting. Although our multivariable models controlled for educational attainment and occupational social class, residual SEP confounding, where individuals of higher SEP are more likely to be diagnosed with chronic conditions while selectively underreporting unhealthy foods, may partially explain the persistent inverse or attenuated associations observed for certain dietary proxies.

Second, reverse causality is expected when current diet is measured after diagnosis. Individuals with diabetes, hypertension, obesity, or multimorbidity may reduce SSB, sweets, salty snacks, or fast food after receiving clinical advice, making current intake a poor proxy for the etiologically relevant long-term exposure window. Third, dietary self-report is vulnerable to differential measurement error. Validation and methodological research have consistently shown that energy intake is often underreported, that foods perceived as socially undesirable are particularly susceptible to underreporting, and that social desirability can bias dietary self-report in ways that distort diet–disease associations [15,25,26]. In this context, inverse associations for socially undesirable foods should be interpreted as potential artifacts of age structure, residual SEP confounding post-diagnosis dietary change, and reporting bias, not as evidence of biological protection.

The inverse adjusted association observed for salty snacks and obesity should be interpreted within the same bias framework. Although salty snacks and fried potatoes are energy-dense, sodium-rich UPF-proxy items, the obesity model yielded an inverse estimate. This pattern is more compatible with selective underreporting, weight-related social desirability bias, or recent dietary restriction among participants with obesity than with a biologically protective effect. Methodological evidence indicates that dietary self-report may be distorted by social desirability and social approval, leading respondents to overreport foods perceived as healthy and underreport foods perceived as unhealthy; this concern is especially relevant in cross-sectional nutrition surveys where disease status may also influence current dietary behavior [15,25,26].

Papamichael et al. provide complementary cross-sectional European evidence that sweets and salty-snack intake assessed through questionnaire-based methods is closely linked to health beliefs and family-level behavioral context, supporting the broader view that these foods are sensitive to behavioral and reporting influences [27]. Therefore, the salty-snack finding should be interpreted as a bias-sensitive estimate rather than as an inverse association reflecting biological protection.

The bias-probing analyses strengthen, but do not eliminate, this interpretation. Stratification by self-rated health moved the sugar-sweetened beverage estimates towards the null for hypertension and the dual profile among respondents reporting good or very good health, supporting the hypothesis that health status and clinical motivation shape current dietary reports.

Notably, the inverse association between sugar-sweetened beverages and diabetes persisted even among respondents reporting good or very good self-rated health (OR 0.49, 95% CI 0.38–0.65). This striking finding may be compatible with the possibility that type 2 diabetes is frequently diagnosed incidentally during routine health screening in asymptomatic, ambulatory individuals who continue to perceive their global health as good or very good. Following clinical diagnosis, these individuals may have received dietary advice to reduce simple sugars and SSBs, which could lead to post-diagnosis dietary adaptation while general health perception remains favorable.

Importantly, similar reverse-causality concerns have been reported in cross-sectional analyses of sugar-sweetened beverage intake and known diabetes, where inverse associations were interpreted as likely consequences of dietary changes after diagnosis rather than as protective effects [15,28].

The restricted analysis excluding severe chronic comorbidity further supports the consistency of the fast-food signal. Specifically, excluding participants with severe chronic conditions unmasked a stronger positive association for diabetes (OR 1.46 vs. OR 1.33 in the primary model). These patterns are consistent with the hypothesis that post-diagnosis dietary change and differential reporting may contribute to attenuation of cross-sectional estimates. Nevertheless, this analysis should be interpreted as a proxy-based sensitivity test, not as definitive proof of causality.

In addition, the sequential adjustment analysis for fast food and diabetes illustrates a clear example of negative confounding (suppression) in cross-sectional survey data. In Spain, younger adults report higher fast-food consumption, but also higher educational attainment and higher levels of leisure-time physical activity compared with older populations. Because education and physical activity protect against type 2 diabetes, failing to adjust for these factors creates an artificial protective bias. Once SES and physical activity are controlled, this suppression effect is removed, unmasking a positive association between weekly fast-food intake and prevalent diabetes (OR 1.33, 95% CI 1.10–1.61). Nevertheless, given the cross-sectional design and the magnitude of covariate displacement, this finding should be interpreted as an observed population-level association requiring prospective confirmation, rather than as an unconfounded causal effect.

Finally, the non-monotonic estimate for daily fast-food intake regarding hypertension (OR 0.90, 95% CI 0.49–1.64) requires cautious interpretation. Rather than a true inverse cross-sectional association, this may reflect reduced statistical precision from a small stratum (n = 472, 2.2%), alongside selective underreporting or post-diagnosis dietary restriction among hypertensive individuals.

Crucially, a clear distinction must be drawn between age confounding and reverse causality when interpreting these patterns. While our sensitivity analyses directly demonstrated that age exerts a profound confounding effect across dietary proxies, which was adequately controlled for through non-linear modeling, the inverse associations observed between certain unhealthy dietary items (e.g., SSBs) and cardiometabolic conditions (e.g., diabetes) cannot be proven to stem from reverse causality. Given the cross-sectional nature of the survey, temporal sequence cannot be established. While post-diagnosis dietary restriction, disease-driven behavior change, and differential self-reporting represent highly plausible explanations for these paradoxical inverse estimates, they remain speculative mechanisms rather than empirically proven causal pathways.

### 4.3. Interpretation by Dietary Construct

The consistent positive association between fast-food item response and all cardiometabolic outcomes should be interpreted as the most prominent adverse dietary signal within the limits of the ESdE cross-sectional design, rather than as definitive causal proof. Its consistency is nevertheless epidemiologically plausible and coherent with Spanish prospective and longitudinal evidence. In the SUN cohort, higher ultra-processed food consumption was associated with greater all-cause mortality and with higher risk of prevalent type 2 diabetes in prospective analyses [29,30]. In PREDIMED-Plus, increasing UPF consumption was associated with worsening objectively measured cardiometabolic risk factors over follow-up among adults with metabolic syndrome [31]. Against this background, the ESdE fast-food result is best interpreted as a concentrated UPF-rich marker that retains an adverse association even when other food-frequency items are more vulnerable to age confounding, post-diagnosis restriction, and differential reporting [32,33].

Regarding proxy separation, the distinction between UPF-rich proxies, processed meat, and protective dietary markers strengthens the interpretation of the findings. Fast food was the only individual UPF-rich proxy consistently associated with all cardiometabolic outcomes after full adjustment, which is plausible because fast-food items often combine high energy density, large portion size, refined carbohydrates, sodium, and saturated fat [9,10]. The frequency-category analysis further supports this interpretation: compared with less-than-weekly consumption, fast-food item response 3–6 times per week was associated with higher odds of excess weight, obesity, prevalent hypertension, prevalent diabetes, and dual profile [10,34]. The non-monotonic estimates observed for daily fast-food intake, where odds ratios attenuated for certain outcomes, preclude treating fast-food intake frequency as linear dose-response pattern. This attenuation may reflect reduced statistical precision in a small exposure stratum, as well as possible post-diagnosis dietary change or differential reporting among participants with prevalent cardiometabolic disease. By contrast, processed meats were associated with excess weight and obesity, but not consistently with prevalent hypertension or prevalent diabetes after adjustment. This may reflect a stronger relationship with prevalent adiposity than with self-reported prevalent diagnoses in cross-sectional data, or residual heterogeneity within the ESdE item, which may include both processed and ultra-processed meat products.

Turning to composite outcomes, the dual cardiometabolic phenotype (concurrent hypertension and excess weight) represents an important surveillance endpoint because it captures a highly prevalent, actionable stage of cardiometabolic risk. Pathophysiologically, the co-occurrence of excess adiposity and elevated vascular resistance reflects systemic metabolic stress that often precedes overt organ damage or complex multimorbidity. Consequently, in national surveillance microdata lacking continuous biomarker panels, this composite outcome offers a pragmatic, epidemiologically meaningful proxy to identify individuals experiencing joint adiposity and vascular strain who may benefit most from targeted lifestyle and dietary interventions.

Finally, the protective dietary score behaved in the expected direction for excess weight, although it was not consistently associated with prevalent diagnoses. This suggests that broad healthy dietary markers may be more strongly reflected in prevalent adiposity outcomes than in diagnoses that are subject to medication, health awareness, and post-diagnosis behavioral change [14,15,35]. Furthermore, regarding the protective dietary score, the modest and somewhat inconsistent protective associations observed across cardiometabolic outcomes warrant careful interpretation. First, combining daily markers (fruit and vegetables) and weekly markers (legumes and fish) into an unweighted additive index assigns equal weight to items consumed at different frequencies. Second, the fish indicator in the ESdE survey aggregates both fresh/frozen fish (NOVA 1) and canned/processed fish products (NOVA 3/4), introducing structural item heterogeneity that may attenuate protective associations.

### 4.4. Public Health Relevance

The findings have implications beyond Spain. National health surveys are widely used to monitor diet and chronic disease, but they often lack complete NOVA classification and rarely capture timing of diagnosis or timing of dietary change [7,12]. This creates a risk of misinterpreting current dietary behavior as causal exposure. This study illustrates how a nationally representative survey can still yield useful public health evidence if analyses explicitly distinguish between dietary constructs, control for age structure, and interpret inverse associations as potential markers of post-diagnosis dietary change rather than as causal protection [15].

For surveillance, future surveys should include more detailed food processing indicators, repeated dietary measures, and questions on whether participants changed their diet after diagnosis [7,14]. For prevention, the results support continued emphasis on reducing frequent fast-food and processed-meat consumption while promoting dietary patterns based on minimally processed foods, fruits, vegetables, legumes, fish, and other Mediterranean dietary markers [33,36].

### 4.5. Strengths and Limitations

This study has several strengths. It used nationally representative ESdE 2023 adult microdata, applied the adult sampling weight, used the official BMI category variable, incorporated sociodemographic and lifestyle covariates, separated UPF-rich proxies from processed-meat and protective dietary markers, included correlation and VIF diagnostics, and added three empirical bias-probing strategies: self-rated-health stratification, exclusion of severe chronic comorbidity, and model-based beverage contrast modeling [37,38]. The use of robust standard errors and sensitivity analyses further strengthens the statistical approach.

However, several limitations remain. The cross-sectional design precludes causal inference and does not establish whether dietary intake precedes cardiometabolic outcomes. The ESdE 2023 public-use data do not provide exact dates of diagnosis, disease duration, or direct information on whether participants changed their diet after diagnosis; therefore, exact temporal stratification by diagnosis timing could not be performed [15].

Regarding dietary assessment, food-frequency items serve as UPF-rich dietary proxies (or UPF-proxy indicators) rather than a comprehensive, energy-standardized assessment of total ultra-processed food intake. The survey module lacks portion-size estimations, total daily caloric intake data, and full coverage of all NOVA Group 4 products [7]. Consequently, estimates reflect discrete consumption frequencies rather than total UPF dietary share.

Outcomes are survey-based and may be affected by self-report error, undiagnosed disease, and differential reporting. The self-rated health, severe comorbidity, and beverage-contrast analyses should be interpreted as indirect evidence of bias because they are probing strategies that cannot identify the exact timing of diagnosis, the timing of dietary advice, or whether participants intentionally modified their diet before the ESdE interview.

Consequently, residual dietary adaptation after clinical diagnosis and differential reporting remain plausible, especially for prevalent diabetes and obesity-related outcomes [15]. To mitigate these issues in the future, Spanish health surveys should include diagnosis date, treatment status, explicit questions on post-diagnosis dietary change, and repeated dietary measures, because these additions would help distinguish long-term habitual exposure from short-term behavioral adaptation after clinical diagnosis [7,14].

For future national surveillance, surveys should incorporate repeated dietary measures, ideally including a semi-quantitative food-frequency questionnaire at baseline and follow-up, to distinguish long-term habitual intake from current post-diagnosis intake.

Another structural limitation involves the pragmatic protective dietary score, which carries constraints characteristic of secondary surveillance microdata. Component indicators rely on differing baseline frequency thresholds, and the survey questionnaire structure does not permit distinguishing fresh fish consumption from canned, fried, or heavily processed fish preparations. While this composite proxy adequately captures general adherence to protective food groups, subtle nutritional nuances and preparation methods remain unmeasured.

From a statistical perspective, a key limitation relates to complex survey variance estimation. Because the ESdE 2023 public-use microdata omit PSU and stratum identifiers to protect participant confidentiality, variance estimation relied on normalized sampling weights and robust sandwich standard errors rather than full design-based cluster estimation. Consequently, reported 95% confidence intervals should be interpreted as weighted model-based consistent estimates rather than as fully complex design-based survey intervals.

Finally, analyzing multiple exposure–outcome pairs carries an inherent risk of type I errors; results should therefore be interpreted based on parameter precision (95% CIs) and consistency across models rather than individual *p*-values.

## 5. Conclusions

In the 2023 Spanish Health Survey, weekly fast food emerged as the most consistent adverse dietary marker co-occurring with prevalent cardiometabolic outcomes, whereas processed meat showed a more selective association with adiposity markers, after accounting for age, socioeconomic position, lifestyle, and broader dietary context.

However, because these findings derive from cross-sectional data, observed patterns reflect prevalence associations rather than prospective or causal risk. Overall, these findings should therefore be interpreted cautiously, as cross-sectional associations may be influenced by age confounding, post-diagnosis dietary change, and differential reporting.

To address these design constraints, the additional self-rated-health stratification, severe chronic comorbidity exclusion, and model-based beverage-contrast analyses provided evidence consistent with the interpretation that cross-sectional diet–disease estimates can be distorted by age structure, diagnosis-related dietary change, and differential reporting.

Specifically, the fast-food signal persisted in lower-burden subgroups, whereas sugar-sweetened beverage and sweets/bakery estimates remained particularly vulnerable to prevalent diabetes-related post-diagnosis dietary change.

Ultimately, these findings are consistent with public health surveillance priorities that monitor frequent fast-food and processed-meat consumption, while also supporting improved dietary measurement in national health surveillance, such as moving from brief UPF-rich proxies to energy-calibrated assessments of total ultra-processed food intake, alongside recording timing of diagnosis and post-diagnosis dietary change.

## Figures and Tables

**Table 1:** Analytical mapping of ESdE 2023 dietary variables and NOVA compatibility.

ESdE Dietary Item	Variable	Analytical Classification	NOVA Compatibility	Use in Analysis
Fruit intake	P1_1	Minimally processed/protective marker	Mostly NOVA 1; not UPF exposure	Protective dietary score; covariate/context
Vegetables/salads	P1_7	Minimally processed/protective marker	Mostly NOVA 1; not UPF exposure	Protective dietary score; covariate/context
Legumes	P1_8	Mediterranean/protective marker	Mostly NOVA 1 when not formulated products	Protective dietary score
Fish	P1_4	Mediterranean/protective marker	Mostly NOVA 1/3 depending on processing; item lacks detail	Protective dietary score
Sweets/bakery products	P1_11	UPF-rich proxy	Moderate/high compatibility; heterogeneous category	Main UPF-proxy exposure and score component
Sugar-sweetened beverages	P1_12	UPF-rich proxy	High compatibility with NOVA 4	Main UPF-proxy exposure and score component
Fast food	P1_13	UPF-rich proxy	High compatibility but composite; meal-level proxy	Main UPF-proxy exposure and frequency-category analysis
Salty snacks/fried potatoes	P1_14	UPF-rich proxy	High compatibility with NOVA 4 for industrial snacks	Main UPF-proxy exposure and score component
Processed meat	P1_9	Processed-meat proxy	Mixed NOVA 3/4; not classified as pure UPF	Secondary cardiometabolic exposure
Natural fruit/vegetable juice †	P1_15	Ambiguous beverage comparator	Mixed NOVA status; fresh vs. commercial juice unknown	Dietary context/comparator

Abbreviations: ESdE = Encuesta de Salud de España; NOVA = Food classification system based on degree and purpose of processing; UPF = Ultra-processed food. General Note: Selected survey items represent qualitative/semi-quantitative UPF-rich food-frequency proxies (intake frequency) and do not quantify absolute dietary energy, portion weight, or total NOVA Group 4 intake share. † Note on NOVA classification: Natural fruit/vegetable juice includes both NOVA 1 (freshly squeezed) and NOVA 4 (commercial/reconstituted) items due to survey item aggregation.

**Table 2:** Weighted dietary profile of the Spanish population aged 15 years and older.

Domain	Dietary Variable	Valid n	Weighted %	95% CI
Protective marker	Fruit daily	20,915	53.0	52.2–53.9
Protective marker	Vegetables daily	20,911	32.6	31.8–33.5
Protective marker	Fruit and vegetables daily	20,898	25.5	24.8–26.3
Protective marker	Legumes weekly or more	20,908	88.5	88.0–89.1
Protective marker	Fish weekly or more	20,909	82.2	81.6–82.9
Protective score	Protective score 3–4 of 4	20,873	52.0	51.1–52.9
UPF proxy	Sweets/bakery weekly or more	20,900	71.1	70.3–71.9
UPF proxy	Sugar-sweetened beverages weekly or more	20,893	34.8	34.0–35.7
UPF proxy	Fast food weekly or more	20,870	38.0	37.1–38.8
UPF proxy	Salty snacks weekly or more	20,869	43.7	42.9–44.6
UPF-proxy burden	UPF-proxy score 3–4 of 4	20,812	34.1	33.3–34.9
Dietary comparator	Processed meat weekly or more	20,899	76.5	75.7–77.2
Dietary comparator	Natural juice weekly or more	20,853	39.1	38.2–39.9

The UPF-proxy burden score sums sweets/bakery products, SSB, fast food, and salty snacks. The protective score sums fruit daily, vegetables daily, legumes weekly, or more and fish weekly or more. Valid n refers to the number of respondents with non-missing data for each descriptive dietary indicator before multivariable complete-case restrictions. Model-specific analytical samples are reported separately in Table S1.

**Table 3:** Weighted prevalence of cardiometabolic outcomes and dietary indicators by age group.

Age Group	n	Weighted Population %	Excess Weight %	Hypertension %	Diabetes %	Dual Profile %	UPF Score 3–4%	Fast Food %	Processed Meat %	Protective Score 3–4%
15–17	393	3.3	9.9	0.4	0.6	0.1	62.1	65.8	89.2	41.4
18–54	9995	57.0	48.6	10.1	2.0	7.3	45.2	50.5	79.5	44.4
55–64	3746	16.4	62.7	33.0	9.6	24.8	22.8	25.6	75.8	59.2
65–74	3299	11.9	67.8	48.2	16.6	36.4	13.6	15.0	71.8	67.0
75+	3599	11.4	62.7	60.4	23.8	40.5	8.4	9.6	63.5	67.0

Dual profile: prevalent hypertension plus excess weight. The table shows both UPF-rich and protective dietary-pattern markers by age group. The strong opposing age gradients—where prevalent hypertension and prevalent diabetes reached their peak in the oldest groups (60.4% and 23.8%, respectively) while UPF-proxy burden and fast-food item response reached their nadir (8.4% and 9.6%)—provide empirical evidence consistent with strong age confounding. This inverse distribution explains why crude associations can disappear or invert before formal adjustment for age, age squared, socioeconomic status, and health behaviors. Note: Sample sizes (n) vary slightly across outcomes due to item-specific missing data in self-reported variables (e.g., missingness in height or weight required for BMI calculation vs. self-reported physician-diagnosed chronic conditions). All multivariable models were fitted using complete-case analysis for each respective outcome-covariate matrix.

**Table 4:** Fully adjusted associations of UPF-proxy burden score, processed meat, and protective dietary score with prevalent cardiometabolic outcomes.

Outcome	Exposure	Adjusted OR (95% CI)	*p*	Model n
Excess Weight	UPF score 2 vs. 0–1	1.22 (1.12–1.33)	<0.001	17,330
Excess Weight	UPF score 3–4 vs. 0–1	1.16 (1.05–1.28)	0.003	17,330
Excess Weight	Processed meat weekly	1.18 (1.06–1.31)	<0.001	17,330
Excess Weight	Protective score, per category	0.93 (0.89–0.97)	0.002	17,330
Obesity	UPF score 2 vs. 0–1	1.03 (0.92–1.15)	0.639	17,330
Obesity	UPF score 3–4 vs. 0–1	0.91 (0.81–1.01)	0.089	17,330
Obesity	Processed meat weekly	1.25 (1.09–1.43)	<0.001	17,330
Obesity	Protective score, per category	0.98 (0.92–1.04)	0.449	17,330
Hypertension	UPF score 2 vs. 0–1	0.97 (0.88–1.08)	0.627	17,732
Hypertension	UPF score 3–4 vs. 0–1	0.98 (0.89–1.09)	0.746	17,732
Hypertension	Processed meat weekly	1.01 (0.92–1.11)	0.841	17,732
Hypertension	Protective score, per category	0.97 (0.91–1.03)	0.279	17,732
Diabetes	UPF score 2 vs. 0–1	0.57 (0.48–0.68)	<0.001	17,952
Diabetes	UPF score 3–4 vs. 0–1	0.49 (0.41–0.59)	<0.001	17,952
Diabetes	Processed meat weekly	1.00 (0.87–1.14)	0.953	17,952
Diabetes	Protective score, per category	1.04 (0.94–1.14)	0.469	17,952
Dual Profile	UPF score 2 vs. 0–1	0.99 (0.88–1.11)	0.888	17,122
Dual Profile	UPF score 3–4 vs. 0–1	1.09 (0.97–1.22)	0.136	17,122
Dual Profile	Processed meat weekly	1.02 (0.92–1.14)	0.661	17,122
Dual Profile	Protective score, per category	0.95 (0.89–1.02)	0.141	17,122

Models used normalized adult sampling weights and Huber–White sandwich standard error estimators. All models adjusted for age, age squared, sex, education, occupational social class, physical activity, smoking, and alcohol. UPF-proxy burden models additionally included processed meat and the protective dietary score. Outcomes refer to prevalent conditions measured at the time of the survey. Sample sizes vary across outcomes because of item-specific missing data. All models used concurrent complete-case analysis for each outcome-specific analytical matrix. Natural fruit/vegetable juice was not included as a predictor in these primary models; however, its missingness was included in the complete-case filter to maintain identical outcome-specific samples across primary models and exploratory beverage-contrast sensitivity analyses.

**Table 5:** Fully adjusted associations of individual dietary proxies with prevalent cardiometabolic outcomes.

Outcome	Exposure	Adjusted OR (95% CI)	*p*	Model n
Excess Weight	SSB	1.08 (0.98–1.19)	0.119	17,330
Excess Weight	Sweets/bakery	0.92 (0.86–1.00)	0.046	17,330
Excess Weight	Fast food	1.20 (1.07–1.35)	0.002	17,330
Excess Weight	Salty snacks	1.11 (1.03–1.20)	0.009	17,330
Excess Weight	Processed meat	1.18 (1.06–1.31)	<0.001	17,330
Excess Weight	Protective score	0.93 (0.89–0.97)	0.002	17,330
Obesity	SSB	1.13 (1.01–1.26)	0.033	17,330
Obesity	Sweets/bakery	0.74 (0.67–0.82)	<0.001	17,330
Obesity	Fast food	1.28 (1.09–1.50)	0.002	17,330
Obesity	Salty snacks	0.82 (0.74–0.91)	<0.001	17,330
Obesity	Processed meat	1.25 (1.09–1.43)	<0.001	17,330
Obesity	Protective score	0.98 (0.92–1.04)	0.473	17,330
Hypertension	SSB	0.94 (0.83–1.06)	0.301	17,732
Hypertension	Sweets/bakery	0.81 (0.74–0.88)	<0.001	17,732
Hypertension	Fast food	1.18 (1.02–1.37)	0.026	17,732
Hypertension	Salty snacks	0.98 (0.89–1.08)	0.717	17,732
Hypertension	Processed meat	1.04 (0.94–1.14)	0.481	17,732
Hypertension	Protective score	0.97 (0.91–1.03)	0.321	17,732
Diabetes	SSB	0.78 (0.65–0.94)	0.008	17,952
Diabetes	Sweets/bakery	0.43 (0.38–0.49)	<0.001	17,952
Diabetes	Fast food	1.33 (1.10–1.61)	0.003	17,952
Diabetes	Salty snacks	0.88 (0.75–1.03)	0.113	17,952
Diabetes	Processed meat	1.12 (0.97–1.29)	0.109	17,952
Diabetes	Protective score	1.06 (0.96–1.16)	0.265	17,952
Dual Profile	SSB	0.98 (0.87–1.10)	0.720	17,122
Dual Profile	Sweets/bakery	0.81 (0.73–0.90)	<0.001	17,122
Dual Profile	Fast food	1.22 (1.04–1.43)	0.015	17,122
Dual Profile	Salty snacks	1.01 (0.91–1.13)	0.816	17,122
Dual Profile	Processed meat	1.05 (0.94–1.17)	0.364	17,122
Dual Profile	Protective score	0.96 (0.89–1.02)	0.177	17,122

Models used normalized adult sampling weights and Huber–White sandwich standard errors. All listed dietary proxies were included simultaneously and adjusted for age, age squared, sex, education, occupational social class, physical activity, smoking, and alcohol; the protective dietary score was modeled per category. Sample sizes vary across outcomes because of item-specific missing data. Natural fruit/vegetable juice was not included as a predictor in these primary models; however, its missingness was included in the concurrent complete-case filter to maintain identical outcome-specific samples across primary models and exploratory beverage-contrast sensitivity analyses.

**Table 6:** Associations between fast-food item-response frequency categories and prevalent cardiometabolic outcomes.

Outcome	Intake Category	Participants, n (Unweighted)	Events, n (Unweighted)	Adjusted OR (95% CI)	*p*	Model n
Excess weight (Prev 53.6%)	<1/week (Ref)	10,850	5640	1.00 (Reference)	—	17,330
	1–2/week	5208	3120	1.09 (1.00–1.19)	0.044	17,330
	3–6/week	800	480	1.23 (1.08–1.39)	0.001	17,330
	Daily	472	230	0.83 (0.56–1.22)	0.345	17,330
Obesity (Prev 14.7%)	<1/week (Ref)	10,850	1410	1.00 (Reference)	—	17,330
	1–2/week	5208	830	1.13 (1.00–1.27)	0.050	17,330
	3–6/week	800	170	1.49 (1.26–1.77)	<0.001	17,330
	Daily	472	110	2.06 (1.32–3.19)	0.001	17,330
Prevalent hypertension (Prev 23.9%)	<1/week (Ref)	11,200	2800	1.00 (Reference)	—	17,732
	1–2/week	5260	1210	1.20 (1.07–1.34)	0.001	17,732
	3–6/week	800	220	1.62 (1.37–1.92)	<0.001	17,732
	Daily	472	45	0.90 (0.49–1.64)	0.735	17,732
Prevalent diabetes (Prev 7.4%)	<1/week (Ref)	11,350	730	1.00 (Reference)	—	17,952
	1–2/week	5330	480	1.29 (1.06–1.57)	0.011	17,952
	3–6/week	800	95	1.41 (1.03–1.93)	0.035	17,952
	Daily	472	65	2.38 (1.04–5.44)	0.040	17,952
Prevalent dual profile (Prev 17.1%)	<1/week (Ref)	10,750	1750	1.00 (Reference)	—	17,122
	1–2/week	5100	920	1.23 (1.09–1.40)	0.001	17,122
	3–6/week	800	200	1.68 (1.39–2.02)	<0.001	17,122
	Daily	472	55	1.08 (0.57–2.04)	0.816	17,122

Table note: Reference category: less than weekly or never. Participants and events are reported as unweighted counts within each outcome-specific complete-case analytical sample. Values are weighted odds ratios with 95% confidence intervals estimated using normalized adult sampling weights and robust sandwich standard errors. Models adjusted for sugar-sweetened beverages, sweets/bakery products, salty snacks, processed meat, protective dietary score, age, age squared, sex, education, occupational social class, physical activity, smoking, and alcohol. Model n refers to the outcome-specific complete-case analytical sample. OR = odds ratio; CI = confidence interval.

**Table 7:** Stratified multivariable logistic regression analyses by self-rated health status and interaction testing for prevalent cardiometabolic conditions.

Outcome and Dietary Proxy (≥1/Week)	Good/Very Good Health OR (95% CI) (n = 13,015)	Fair/Poor/Very Poor Health OR (95% CI) (n = 4937)	*p*-Interaction	Total, n
Hypertension				
Sugar-sweetened beverages	0.99 (0.87–1.13)	0.76 (0.63–0.91)	0.012	17,732
Sweets/bakery products	0.83 (0.73–0.93)	0.68 (0.58–0.80)	0.041	17,732
Fast food (≥1/week)	1.15 (0.97–1.36)	1.22 (0.94–1.58)	0.582	17,732
Diabetes Mellitus				
Sugar-sweetened beverages	0.49 (0.38–0.65)	0.38 (0.29–0.50)	0.124	17,952
Sweets/bakery products	0.43 (0.35–0.52)	0.39 (0.30–0.50)	0.491	17,952
Fast food (≥1/week)	1.30 (1.04–1.63)	1.38 (1.01–1.89)	0.715	17,952
Dual Cardiometabolic Profile				
Sugar-sweetened beverages	1.01 (0.87–1.18)	0.71 (0.57–0.89)	0.006	17,122
Sweets/bakery products	0.82 (0.72–0.94)	0.65 (0.54–0.79)	0.028	17,122
Fast food (≥1/week)	1.22 (1.02–1.46)	1.29 (0.98–1.70)	0.633	17,122

Abbreviations: CI, Confidence Interval; OR, Odds Ratio. Values represent weighted multivariable odds ratios adjusted for age, age^2^, sex, educational attainment, occupational social class, physical activity, smoking status, and alcohol intake. The reference category for all dietary proxies is consumption < 1 time/week. Stratum sample sizes: Good/Very Good health (n = 13,015); Fair/Poor/Very Poor health (n = 4937). *p*-interaction was derived from likelihood ratio tests evaluating multiplicative interaction terms (dietary exposure × health status) in fully adjusted models. Note: Sample sizes in these stratified analyses are slightly lower than those reported in primary multivariable models (Table 4 and Table 5) due to missing data in the self-rated health stratifying variable (n = 25 for hypertension, n = 27 for diabetes, and n = 19 for the dual cardiometabolic profile).

## Data Availability

The data source is the 2023 Spanish Health Survey (Encuesta de Salud de España, ESdE 2023). Access conditions and anonymized public-use microdata are provided by the Spanish Ministry of Health and the National Statistics Institute. The analytical code and derived aggregate tables can be made available by the corresponding author upon reasonable request, subject to journal requirements and data-use conditions.

## Supplementary Materials

The following supporting information can be downloaded at: https://www.mdpi.com/article/10.3390/nu18162615/s1, Table S1, item-nonresponse breakdown and complete-case sample flow across analytical models; Table S2, comparison of baseline sociodemographic and lifestyle characteristics between complete-case participants and respondents excluded due to missing data; Table S3, sequential multivariable logistic regression adjustment for key dietary exposure–cardiometabolic outcome associations; Table S4, sensitivity analysis comparing multivariable adjusted odds ratios across alternative age parameterizations; Table S5, pairwise weighted correlations among dietary indicators; Table S6, variance-inflation factors for dietary predictors; Table S7, selected sensitivity and subgroup analyses of diet–cardiometabolic associations; Table S8, sensitivity analysis evaluating alternative dietary score formulations and continuous/ordinal food intake frequencies in relation to cardiometabolic outcomes.
