# Supplementary material for: Ultra-Processed Food-Rich Dietary Proxies, Dietary-Pattern Context, and Cardiometabolic Outcomes in Spain: Weighted Evidence Consistent with Age Confounding and Possible Reverse-Causality Bias from the 2023 Spanish Health Survey
Source: Nutrients. 2026 Aug 10;18(16):2615. doi: 10.3390/nu18162615 (PMC13516521; doi:10.3390/nu18162615)
Supplement: Supplementary file 1 [file nutrients-18-02615-s001.zip › nutrients-4456812-supplementary.pdf]

Table S1. Item-nonresponse breakdown and complete-case sample flow across analytical models (2023 Spanish Health Survey, n=21,032)

Category / Variable / Model	Missing / Excluded (n)	Missing / Excluded (%)	Complete-Case Sample (n)	Sample Coverage (%)
**Outcomes**				
**Height / Weight (BMI calculation)**	3,302	15.7%	17,730	84.3%
**Arterial Hypertension diagnosis**	288	1.4%	20,744	98.6%
**Diabetes Mellitus diagnosis**	68	0.3%	20,964	99.7%
**Dual Profile (Hypertension + Excess weight)**	3,510	16.7%	17,522	83.3%
**Dietary Exposures / Descriptive dietary indicators**				
**Sweets and bakery products**	132	0.6%	20,900	99.4%
**Sugar-sweetened beverages (SSB)**	139	0.7%	20,893	99.3%
**Fast food consumption frequency**	162	0.8%	20,870	99.2%
**Salty snacks / fried potatoes**	163	0.8%	20,869	99.2%
**Processed meat frequency**	133	0.6%	20,899	99.4%
**UPF-proxy burden score**	220	1.0%	20,812	99.0%
**Protective dietary score**	159	0.8%	20,873	99.2%
**Natural fruit/vegetable juice**	179	0.9%	20,853	99.1%
**Covariates**				
**Educational level**	142	0.7%	20,890	99.3%
**Occupational social class**	985	4.7%	20,047	95.3%
**Leisure-time physical activity**	312	1.5%	20,720	98.5%
**Smoking status**	118	0.6%	20,914	99.4%
**Alcohol intake frequency**	285	1.4%	20,747	98.6%
**Fully Adjusted Multivariable Models**				
**Excess Weight Model**	3,702	17.6%	17,330	82.4%
**Obesity Model**	3,702	17.6%	17,330	82.4%
**Hypertension Model**	3,300	15.7%	17,732	84.3%
**Diabetes Model**	3,080	14.6%	17,952	85.4%
**Dual Profile Model (Hypertension + Excess weight)**	3,910	18.6%	17,122	81.4%

Table note: Sample flow based on the 2023 Spanish Health Survey (ESdE adult microdata, n=21,032). Item non-response across individual variables is non-cumulative because single respondents may have missing values across multiple indicators simultaneously. The final multivariable regression models reflect a concurrent complete-case approach. To ensure strict internal consistency between the primary models and the exploratory beverage-contrast sensitivity analyses, the analytical denominator was restricted to participants with non-missing data across the relevant outcome, all primary dietary exposures, all adjustment covariates, processed meat, protective dietary score, and the natural fruit/vegetable juice variable. Natural fruit/vegetable juice was not included as a predictor in the primary models reported in Tables 4 and 5, but missingness in this variable was included in the complete-case filtering step. This design ensured that primary and beverage-contrast estimates were derived from the same outcome-specific analytical populations.

Table S2. Comparison of baseline sociodemographic and lifestyle characteristics between included complete-case participants and respondents excluded due to missing data (2023 Spanish Health Survey, n=21,032).

**Baseline Characteristic**	**Included Participants (n=17,330)**	**Excluded Participants (n=3,702)**	**p-value / Difference**
**Age (years), Mean (SD)**	52.4 (18.6)	56.1 (20.3)	< 0.001
**Age group, n (%)**			
**15–34 years**	3,622 (20.9%)	621 (16.8%)	
**35–49 years**	4,215 (24.3%)	710 (19.2%)	
**50–64 years**	4,510 (26.0%)	882 (23.8%)	< 0.001
**≥ 65 years**	4,983 (28.8%)	1,489 (40.2%)	
**Sex, n (% Female)**	9,120 (52.6%)	2,110 (57.0%)	< 0.001
**Educational level, n (%)**			
**Primary / Lower**	4,112 (23.7%)	1,421 (38.4%)	
**Secondary**	7,625 (44.0%)	1,320 (35.7%)	< 0.001
**Higher / Tertiary**	5,593 (32.3%)	961 (26.0%)	
**Occupational Social Class, n (%)**			
**Class I–II (High)**	4,210 (24.3%)	582 (15.7%)	
**Class III–IV (Medium)**	7,125 (41.1%)	1,210 (32.7%)	< 0.001
**Class V–VI (Low)**	5,995 (34.6%)	1,910 (51.6%)	
**Smoking Status, n (% Current smoker)**	3,421 (19.7%)	592 (16.0%)	< 0.001
**Self-rated Health n (% Good / Very good)**	12,299 (71.0%)	2,102 (56.8%)	< 0.001

Table note. Values are unweighted counts (n) and unweighted sample percentages (%). Sample flow based on the 2023 Spanish Health Survey (Encuesta Nacional de Salud / ESdE adult microdata, n=21,032). Differences between the included complete-case sample (n=17,330) and participants excluded due to missing data in multivariable models (n=3,702) were evaluated using Pearson's Chi-square tests for categorical variables and Student's t-test for continuous variables. Statistically significant differences ($p < 0.05$) indicate non-random patterns of missingness across sociodemographic and lifestyle characteristics.

Table S3 Sequential multivariable logistic regression adjustment for key dietary exposure–cardiometabolic outcome associations (2023 Spanish Health Survey).

**Dietary Exposure & Outcome**	**Model 0: Crude**	**Model 1: + Age & Sex**	**Model 2: + Socioeconomic Status**	**Model 3: + Lifestyle Factors**	**Model 4: Full Model**
**Fast food (≥ 1/week)**					
Diabetes Mellitus (n=17,952)	0.58 (0.48–0.70)	0.86 (0.71–1.04)	1.09 (0.89–1.33)	1.25 (1.02–1.52)	1.33 (1.10–1.61)
Excess Weight (n=17,330)	0.72 (0.65–0.80)	1.11 (0.99–1.24)	1.16 (1.04–1.30)	1.18 (1.05–1.32)	1.20 (1.07–1.35)
Hypertension (n=17,732)	0.42 (0.36–0.48)	0.93 (0.80–1.07)	1.05 (0.91–1.22)	1.13 (0.98–1.31)	1.18 (1.02–1.37)
**Sugar-sweetened beverages (≥ 1/week)**					
Diabetes Mellitus (n=17,952)	0.46 (0.38–0.54)	0.71 (0.59–0.85)	0.74 (0.61–0.89)	0.77 (0.64–0.93)	0.78 (0.65–0.94)
Excess Weight (n=17,330)	0.76 (0.69–0.83)	1.04 (0.95–1.14)	1.06 (0.96–1.16)	1.07 (0.97–1.18)	1.08 (0.98–1.19)
**Processed meat (≥ 1/week)**					
Excess Weight (n=17,330)	1.12 (1.02–1.23)	1.15 (1.04–1.28)	1.17 (1.05–1.30)	1.17 (1.05–1.30)	1.18 (1.06–1.31)
Obesity (n=17,330)	1.18 (1.04–1.34)	1.21 (1.06–1.38)	1.23 (1.07–1.41)	1.24 (1.08–1.42)	1.25 (1.09–1.43)

Abbreviations: CI, Confidence Interval; OR, Odds Ratio. **Note:** Values represent weighted Odds Ratios with 95% Confidence Intervals [OR (95% CI)] estimated within complete-case analytical samples. Survey sampling weights and Huber–White robust sandwich standard errors were incorporated across all model specifications. Model 0 (Crude): Unadjusted univariable model. Model 1 (+ Age & Sex): Adjusted for age, age squared (age + age^2), and sex. Model 2 (+ Socioeconomic Status): Adjusted for Model 1 covariates plus educational attainment (primary/less, secondary, higher education) and occupational social class (Classes I–VI). Model 3 (+ Lifestyle Factors): Adjusted for Model 2 covariates plus leisure-time physical activity (sedentary, light, moderate/intense), smoking status (never, former, current), and alcohol consumption frequency. Model 4 (Full Model): Adjusted for Model 3 covariates plus broader dietary context (mutually adjusting for other dietary exposure proxies and protective dietary score).

Table S4. Sensitivity analysis comparing multivariable adjusted Odds Ratios (OR) and 95% Confidence Intervals (95% CI) across alternative age parameterizations for primary diet–cardiometabolic associations (2023 Spanish Health Survey).

Outcome / Dietary Exposure	Model 1: Continuous Quadratic Age (age+age2) [Primary Specification]	Model 2: Categorical Age Groups (10-Year Brackets)	Model 3: Restricted Cubic Splines (4 Knots)
**Excess Weight (BMI ≥ 25 kg/m²)**			
**Fast food (≥ 1/week vs < 1/week)**	1.20 (1.07–1.35)	1.19 (1.06–1.34)	1.20 (1.07–1.35)
**Sugar-sweetened beverages (≥ 1/week vs < 1/week)**	1.08 (0.98–1.19)	1.08 (0.97–1.19)	1.08 (0.98–1.19)
**Processed meat (≥ 1/week vs < 1/week)**	1.18 (1.06–1.31)	1.17 (1.05–1.30)	1.18 (1.06–1.31)
**UPF-proxy burden score (High vs. Low)**	1.16 (1.05–1.28)	1.15 (1.04–1.27)	1.16 (1.05–1.28)
**Obesity (BMI ≥ 30 kg/m²)**			
**Fast food (≥ 1/week vs < 1/week)**	1.28 (1.09–1.50)	1.27 (1.08–1.49)	1.28 (1.09–1.50)
**Processed meat (≥ 1/week vs < 1/week)**	1.25 (1.09–1.43)	1.24 (1.08–1.42)	1.25 (1.09–1.43)
**Arterial Hypertension**			
**Fast food (≥ 1/week vs < 1/week)**	1.18 (1.02–1.37)	1.17 (1.01–1.36)	1.18 (1.02–1.37)
**Sugar-sweetened beverages (≥ 1/week vs < 1/week)**	0.94 (0.83–1.06)	0.94 (0.83–1.06)	0.94 (0.83–1.06)
**Diabetes Mellitus**			
**Fast food (≥ 1/week vs < 1/week)**	1.33 (1.10–1.61)	1.32 (1.09–1.60)	1.33 (1.10–1.61)
**Sugar-sweetened beverages (≥ 1/week vs < 1/week)**	0.78 (0.65–0.94)	0.77 (0.64–0.93)	0.78 (0.65–0.94)
**Dual Profile (Hypertension + Excess Weight)**			
**Fast food (≥ 1/week vs < 1/week)**	1.22 (1.04–1.43)	1.21 (1.03–1.42)	1.22 (1.04–1.43)
**UPF-proxy burden score (High vs. Low)**	1.14 (0.99–1.31)	1.13 (0.98–1.30)	1.14 (0.99–1.31)

Table note. Values are weighted multivariable Odds Ratios (OR) and 95% Confidence Intervals (95% CI). All models are estimated within complete-case analytical samples and adjusted for sex, educational level, occupational social class, leisure-time physical activity, smoking status, alcohol consumption frequency, protective dietary score, and mutually adjusted for other dietary exposures. Model 1 specifies age as continuous with a quadratic term (age + age²). Model 2 parameterizes age into 10-year categorical brackets. Model 3 specifies age using restricted cubic splines with 4 knots.

Table S5. Pairwise weigted correlations among dietary indicators

Indicator	UPF score	Sweets	SSB	Fast food	Snacks	Proc. meat	Protective score	Fruit	Vegetables	Legumes	Fish
**UPF score**	1.00	0.51	0.71	0.76	0.75	0.27	-0.16	-0.19	-0.17	0.01	-0.05
**Sweets**	0.51	1.00	0.31	0.27	0.30	0.24	-0.05	-0.07	-0.14	0.05	0.00
**SSB**	0.71	0.31	1.00	0.45	0.39	0.19	-0.15	-0.18	-0.14	0.01	-0.04
**Fast food**	0.76	0.27	0.45	1.00	0.48	0.19	-0.12	-0.17	-0.10	0.02	-0.03
**Snacks**	0.75	0.30	0.39	0.48	1.00	0.23	-0.13	-0.13	-0.14	-0.00	-0.05
**Proc. meat**	0.27	0.24	0.19	0.19	0.23	1.00	-0.01	-0.04	-0.09	0.07	0.05
**Protective score**	-0.16	-0.05	-0.15	-0.12	-0.13	-0.01	1.00	0.72	0.54	0.42	0.58
**Fruit**	-0.19	-0.07	-0.18	-0.17	-0.13	-0.04	0.72	1.00	0.35	0.09	0.16
**Vegetables**	-0.17	-0.14	-0.14	-0.10	-0.14	-0.09	0.54	0.35	1.00	0.08	0.14
**Legumes**	0.01	0.05	0.01	0.02	-0.00	0.07	0.42	0.09	0.08	1.00	0.18
**Fish**	-0.05	0.00	-0.04	-0.03	-0.05	0.05	0.58	0.16	0.14	0.18	1.00

Table S6. Variance-inflation factors for dietary predictors. VIFs were calculated for the main dietary predictors in the individual-proxy multivariable specification. Values did not indicate problematic collinearity among dietary predictors.

Dietary predictor	VIF
**Sweets/bakery weekly**	1.16
**SSB weekly**	1.40
**Fast food weekly**	1.52
**Salty snacks weekly**	1.45
**Processed meat weekly**	1.09
**Protective score ordinal**	1.04

Abbreviations: SSB, sugar-sweetened beverages; VIF, variance-inflation factor; UPF, ultra-processed food.

Table S7. Selected sensitivity and subgroup analyses of diet–cardiometabolic associations.

Analysis / Subgroup	Outcome	Exposure / Indicator	Weighted OR (95% CI)	Model n
**Good/very good self-rated health**	Hypertension	Fast food weekly or more	1.15 (0.97–1.36)	12,299
	Diabetes	SSB weekly or more	0.49 (0.38–0.65)	12,427
	Hypertension + Excess Weight	Fast food weekly or more	1.22 (1.02–1.46)	11,916
**No severe chronic comorbidity**	Excess Weight	Fast food weekly or more	1.09 (1.00–1.19)	14,099
	Obesity	Fast food weekly or more	1.18 (1.04–1.33)	14,099
**(participants with severe diagnoses excluded)**	Hypertension	Fast food weekly or more	1.21 (1.07–1.37)	14,427
	Diabetes	Fast food weekly or more	1.46 (1.17–1.81)	14,618
	Hypertension + Excess Weight	Fast food weekly or more	1.33 (1.16–1.52)	13,920
	Excess Weight	Processed meat weekly or more	1.15 (1.05–1.25)	14,099
	Obesity	Processed meat weekly or more	1.31 (1.15–1.49)	14,099
	Diabetes	Processed meat weekly or more	1.23 (1.02–1.47)	14,618
	Excess Weight	UPF-proxy score 3–4 vs 0–1	1.35 (1.20–1.52)	14,099
	Diabetes	UPF-proxy score 3–4 vs 0–1	1.29 (1.09–1.53)	14,618
**Beverage contrast specification**	Excess Weight	Sugar-sweetened beverages	1.22 (1.10–1.35)	17,330
	Obesity	Sugar-sweetened beverages	1.27 (1.12–1.44)	17,330
	Hypertension	Sugar-sweetened beverages	1.18 (1.05–1.33)	17,732
	Diabetes	Sugar-sweetened beverages	1.34 (1.15–1.56)	17,952
**Natural juice vs. SSB contrast**	Excess Weight	Natural juice vs SSB	0.89 (0.80–0.99)	17,330
	Obesity	Natural juice vs SSB	0.81 (0.70–0.95)	17,330
	Diabetes	Natural juice vs SSB	1.54 (1.22–1.95)	17,952
**Restricted adult sample (excluding 15–17 yrs)**	Excess Weight	Fast food weekly or more	1.20 (1.07–1.35)	16,937
	Obesity	Fast food weekly or more	1.28 (1.09–1.50)	16,937
	Hypertension	Fast food weekly or more	1.18 (1.02–1.37)	17,339
	Diabetes	Fast food weekly or more	1.34 (1.11–1.61)	17,559
	Hypertension + Excess Weight	Fast food weekly or more	1.22 (1.04–1.43)	16,729

Table note. Values are weighted odds ratios with 95% confidence intervals estimated using normalized adult sampling weights and robust sandwich standard errors. Models were adjusted using the same covariate structure as the corresponding fully adjusted main models. In the restricted adult sample analysis, participants aged 15–17 years were excluded.

Table S8. Sensitivity analysis evaluating alternative dietary score formulations and continuous/ordinal food intake frequencies in relation to cardiometabolic outcomes.

Dietary Exposure Specification	Excess Weight (n=17,330)	Obesity (n=17,330)	Hypertension (n=17,732)	Diabetes Mellitus (n=17,952)	Dual Profile (n=17,122)
**Continuous UPF-Proxy Score** ***(per 1-unit increase, 0–4)***	1.07 (1.03–1.11)	1.09 (1.04–1.15)	1.02 (0.97–1.07)	1.04 (0.97–1.11)	1.06 (1.01–1.12)
**Ordinal Intake Frequencies** ***(per category increase)***					
**Fast food frequency**	1.08 (1.03–1.13)	1.11 (1.04–1.18)	1.04 (0.98–1.10)	1.10 (1.01–1.19)	1.07 (1.00–1.14)
**Processed meat frequency**	1.07 (1.02–1.12)	1.10 (1.03–1.17)	1.01 (0.96–1.06)	0.99 (0.92–1.06)	1.03 (0.97–1.09)
**Sugar-sweetened beverages frequency**	1.02 (0.97–1.07)	1.04 (0.98–1.10)	0.97 (0.92–1.02)	0.90 (0.83–0.97)	0.96 (0.90–1.02)
**Continuous Protective Score** ***(per 1-unit increase, 0–4)***	0.96 (0.92–1.00)	0.93 (0.88–0.98)	0.98 (0.93–1.03)	1.01 (0.94–1.08)	0.97 (0.92–1.02)

Abbreviations: CI, Confidence Interval; OR, Odds Ratio; UPF, Ultra-processed food. **Table note.** Values represent weighted multivariable Odds Ratios with 95% Confidence Intervals [OR (95% CI)] estimated within complete-case analytical samples. All models are fully adjusted for age, age squared (age + age^2), sex, educational level, occupational social class, leisure-time physical activity, smoking status, and alcohol consumption frequency. Ordinal frequency categories: 0 = never/rarely, 1 = 1–2 times/month, 2 = 1–2 times/week, 3 = 3–6 times/week, 4 = daily.
